# Transcriptional Correlates of Chronic Alcohol Neuroadaptation in *Drosophila* Larvae

**DOI:** 10.3389/fnbeh.2021.768694

**Published:** 2021-11-04

**Authors:** Amanda Anqueira-González, Jenny P. Acevedo-Gonzalez, Airined Montes-Mercado, Claudia Irizarry-Hernández, Nicolás L. Fuenzalida-Uribe, Alfredo Ghezzi

**Affiliations:** Department of Biology, University of Puerto Rico-Río Piedras Campus, San Juan, PR, United States

**Keywords:** neuroadaptation, alcohol use disorder, *Drosophila melanogaster*, cognition, differential gene expression, RNA-seq

## Abstract

When presented with the choice, *Drosophila melanogaster* females will often prefer to lay eggs on food containing a significant amount of alcohol. While, in some cases, this behavioral decision can provide a survival advantage to the developing larvae, it can also lead to developmental and cognitive problems. Alcohol consumption can affect executive functions, episodic memory, and other brain function capacities. However, in the fruit fly, the initial cognitive effects of alcohol consumption have been shown to reverse upon persistent exposure to alcohol. Using an olfactory conditioning assay where an odorant is implemented as a conditioned stimulus and paired with a heat shock as an unconditioned stimulus, a previous study has shown that when exposed to a short acute dose of alcohol, *Drosophila* larvae can no longer learn this association. Interestingly, upon prolonged chronic alcohol exposure, larvae seem to successfully avoid the conditioned stimulus just as well as control alcohol-naive larvae, suggestive of alcohol-induced neuroadaptations. However, the mechanisms by which *Drosophila* adapt to the presence of alcohol remains unknown. In this study, we explore the transcriptional correlates of neuroadaptation in *Drosophila* larvae exposed to chronic alcohol to understand the genetic and cellular components responsible for this adaptation. For this, we employed RNA sequencing technology to evaluate differences in gene expression in the brain of larvae chronically exposed to alcohol. Our results suggest that alcohol-induced neuroadaptations are modulated by a diverse array of synaptic genes within the larval brain through a series of epigenetic modulators.

## Introduction

The fruit fly *Drosophila melanogaster* has been used for over a hundred years as a model organism to further our understanding of various biological processes. More recently, this versatile model system has also provided insights into the behavioral genetics of alcohol use disorders since its alcohol responses closely resemble mammalian responses (reviewed in Rothenfluh et al., 2014). However, it is important to note that *Drosophila melanogaster* has an intimate relationship with alcohol. They naturally feed on decaying fruits, which contain varying concentrations of alcohol—a by-product of fermentation. Consumption of food with low to moderate levels of alcohol [(alcohol) < 4%) can result in increased larval fitness, while higher concentrations [(alcohol) > 4%] can cause significant developmental delays and cognitive dysfunction (McKenzie and Parsons, 1972; Robinson et al., 2012b; Schumann et al., 2021). Thus, it is not surprising that adult *D. melanogaster* females prefer to lay eggs on foods containing low to moderate alcohol concentrations while avoiding concentrations higher than 5% (Kacsoh et al., 2013). Interestingly, when threatened by parasitic wasps or in certain spatial arrangements, female flies will often choose to lay on food that surpasses concentrations of 10% alcohol by volume (Kacsoh et al., 2013; Sumethasorn and Turner, 2016).

*Drosophila* larvae can adapt to the presence of alcohol in the food and minimize some of its detrimental effects. By employing an olfactory heat shock conditioning assay, a previous study by Robinson et al. (2012a) showed that while *Drosophila* larvae acutely maintained on ethanol food for 1 h failed to establish memories after conditioning, larvae chronically exposed to alcohol over 6 days were capable of establishing a conditioned memory just as well as larvae that had not been exposed to alcohol (Robinson et al., 2012a). This study demonstrated that acute exposure to alcohol results in a deficiency in learning while chronic exposure does not present this deficiency. Consequently, this result indicates that the constant presence of alcohol induces a series of neuroadaptations that mitigate the effects of alcohol on learning.

Cognitive functions including attention, working memory, processing speed, visuospatial abilities, executive functions, impulsivity, learning, memory, and verbal fluency have all been shown to be impaired by alcohol dependence in humans (Stavro et al., 2013). While most of the cognitive repercussions of alcohol consumption are well-documented, the molecular and cellular mechanisms underlying interactions between alcohol neuroadaptations and cognitive impairments remain largely unknown. By employing the use of next-generation sequencing, here we evaluate larval brain differential gene expression between alcohol-adapted larvae and alcohol naive larvae to identify the genes whose expression changes because of these neuroadaptations.

## Methods

### Fly Stocks and Chronic Alcohol Treatment

Wild-type *Drosophila melanogaster* flies of the Canton S strain (CS) were used in all experiments. Flies were raised in standard cornmeal food at 25°C on a 12-h light/dark schedule. Chronic alcohol exposure was performed following the procedure described by Robinson et al. (2012a). Briefly, standard cornmeal food was supplemented with pure alcohol (Ethyl alcohol no. S73985A, Fisher Scientific) to obtain a 5% alcohol concentration in food (alcohol food). After melting the standard fly food for approximately 30 seconds, 2.65 mL of 95% alcohol was added to 50 mL of fly food just before re-solidification. Food was stirred to homogenize the solution and allowed to cool at room temperature for 24 h. After the 24 h were through, adult CS files were transferred to each bottle and allowed to lay eggs for 24 h. At the end of the 24 h, adult flies were discarded. The alcohol food was supplemented daily by pipetting 0.3 mL with 10% alcohol on the surface of the food until larvae reached the third instar stage, ∼5–6 days after eggs had been laid. For control larvae, the food was supplemented with water when melted instead of alcohol (control food). Control food was not supplemented daily.

### Larval Brain Dissection and RNA Extraction

Six days after egg-laying, as larvae began to crawl out of the food to proceed with pupae formation, individual third-instar larvae were collected using a needle, transferred to microcentrifuge tubes, and frozen at −80°C for 24–48 h. Freezing was followed by the addition of 200 μL of RNAlater-ICE Frozen Tissue Transition Solution (AM7030, Thermo Fisher Scientific) to larvae. Approximately 30 larval brains were dissected per replicate in RNAlater-ICE, for a total of three replicates (∼90 larval brains) for each group. Total RNA was extracted from larval brains using the EZ1 RNA Tissue mini kit (EZ1 RNA Tissue Mini Kit no. 959034, Qiagen). All samples were treated with DNase I (DNase Set no. 79254, Qiagen). RNA concentration and quality were evaluated using a Qubit 2.0 fluorometer (Life Technologies, United States) and Agilent 2100 Bioanalyzer (Agilent Technologies, United States).

### RNA Sequencing and Bioinformatic Analyses

Capture of mRNA (poly-A selection) and library preparation was carried out using the Illumina TruSeq^®^ Stranded mRNA Library Prep kit from 300 ng of total RNA. Libraries were sequenced with an Illumina NextSeq 500/550 High Output Kit v2.5 for 80 cycles following the manufacturer’s protocol (Illumina, Inc.). At least 20 million reads were obtained for each replicate (a pool of 30 brains). The generated sequencing data was stored in FASTQ format and, the quality of the sequencing reads was assessed using FastQC 0.11.9. Adapters present in the sequences were eliminated using Trimmomatic 0.39. Tools from the “new tuxedo suite” were used to process the reads following the protocol by Pertea et al. (2016). Briefly, sequence alignment to the *Drosophila* dm6 genome assembly was completed using *HISAT* 2.2.0; *SamTools* was used to compress, sort, and index transcripts, while merging and quantifying transcripts was done using *StringTie* 2.1.4. Calculation of differential expression was achieved using *Ballgown* (Pertea et al., 2016) and visualized using R (Frazee et al., 2015). To remove low abundance genes, we applied a variance filter to remove all transcripts with a variance across samples of <1. No rRNA or ncRNA filtering was performed. For gene ontology (GO) annotation search and enrichment analysis, significant gene categories for differentially expressed genes were identified using DAVID web-accessible version 6.8 using default parameters and official gene symbols as input (Huang da et al., 2009).

## Results

**FIGURE 1:**
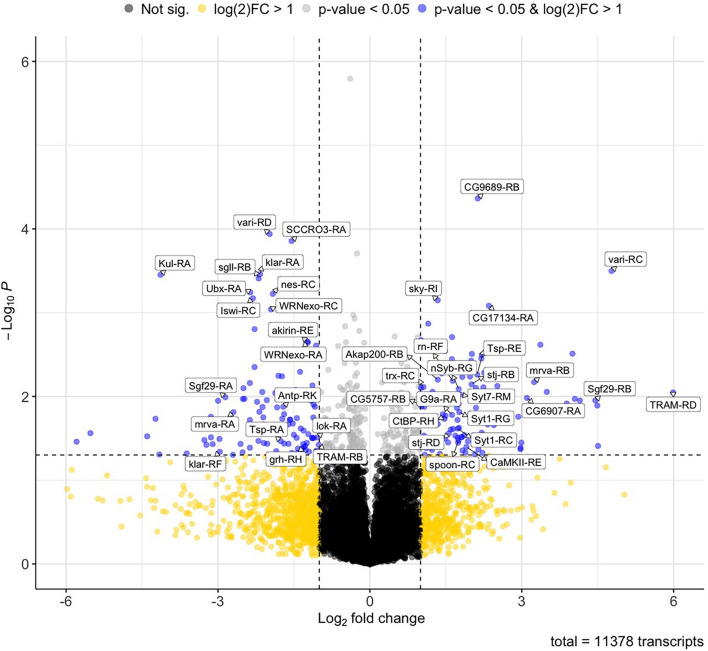
Volcano plot of differentially expressed transcripts induced by chronic alcohol exposure. Volcano plots showing fold change of differential transcript expression [log2 fold change] compared to the inverse of statistical significance [−log_10_ (*P*-value)]. Points in black or yellow are transcripts with no significant changes in expression (*P* > 0.05). Points in gray and blue correspond to transcripts with significant changes in expression (*P* < 0.05). Points in blue correspond to transcripts with significant changes in expression (*P* < 0.05) and that display a fold change higher than 2-fold (log2 fold change > 1). Names of genes encoding representative transcripts are labeled in the plot.

RNA-seq analysis revealed significant changes in the expression of mRNA transcripts between control (alcohol-naive) larvae and the alcohol-grown larvae. Specifically, a total of 216 transcripts were found to be differentially expressed with a *p*-value < 0.05 and a fold change cutoff of 2-fold (log2 > 1). Of these, 113 transcripts were upregulated, while 103 transcripts were downregulated. Differentially expressed transcripts were defined as such using a fold change cutoff of 2-fold (log2 fold change > 1) and a *P*-value < 0.5. The distribution of these transcripts within these categories is shown in Figure 1.

A total of 201 genes were represented in the 216 differentially expressed transcripts, with a total of 15 genes that were represented by two distinct differently expressed transcriptional isoforms. These genes are *CG18649*, *corto*, *Eno*, *Fbp1*, *Gyg*, *klar*, *mrva*, *Sgf29*, *stj*, *Syt1*, *TRAM*, *trol*, *Tsp*, *vari*, and *WRNexo*. Furthermore, it was particularly surprising to observe that of the 15 doubly represented genes, 5 genes had transcripts with opposing alcohol-induced effects, where 1 isoform was significantly upregulated by alcohol and the other significantly downregulated by alcohol, suggesting possible molecular switches. These genes are displayed in Table 1. A complete list of all differentially expressed transcripts, the represented genes is presented in Supplementary Table 1. Similarly, a complete list of FPKM counts for all samples tested is presented in Supplementary Table 2.

**TABLE 1:** Genes with transcripts showing opposing alcohol-induced effects.

**Gene symbol**	**Gene name**	**Differentially expressed Transcript**	**Flybase ID**	**Log2 Fold Change**	***P*-value**	**Effect on gene expression**
*mrva*	Minerva	mrva-RA	FBtr0076884	–2.69	1.54E-02	Downregulated
		mrva-RB	FBtr0332734	3.24	6.73E-03	Upregulated
*Sgf29*	SAGA-associated factor 29 kDa	Sgf29-RA	FBtr0071617	–2.86	1.02E-02	Downregulated
		Sgf29-RB	FBtr0342888	4.45	1.12E-02	Upregulated
*TRAM*	TRAM	TRAM-RB	FBtr0070167	–1.01	3.78E-02	Downregulated
		TRAM-RD	FBtr0307182	5.99	8.95E-03	Upregulated
*Tsp*	Thrombospondin	Tsp-RA	FBtr0079312	–1.76	3.42E-02	Downregulated
		Tsp-RE	FBtr0079314	2.12	5.48E-03	Upregulated
*vari*	Varicose	vari-RD	FBtr0089945	–1.98	1.15E-04	Downregulated
		vari-RC	FBtr0089946	4.77	3.18E-04	Upregulated

*Official gene symbols, gene names, and Flybase IDs (flybase.org) for each gene and corresponding transcripts are shown along with Log2 fold change and *P*-values. Positive log2 fold change values indicate alcohol-induced upregulation, whereas negative values indicate alcohol-induced downregulation.*

**FIGURE 2:**
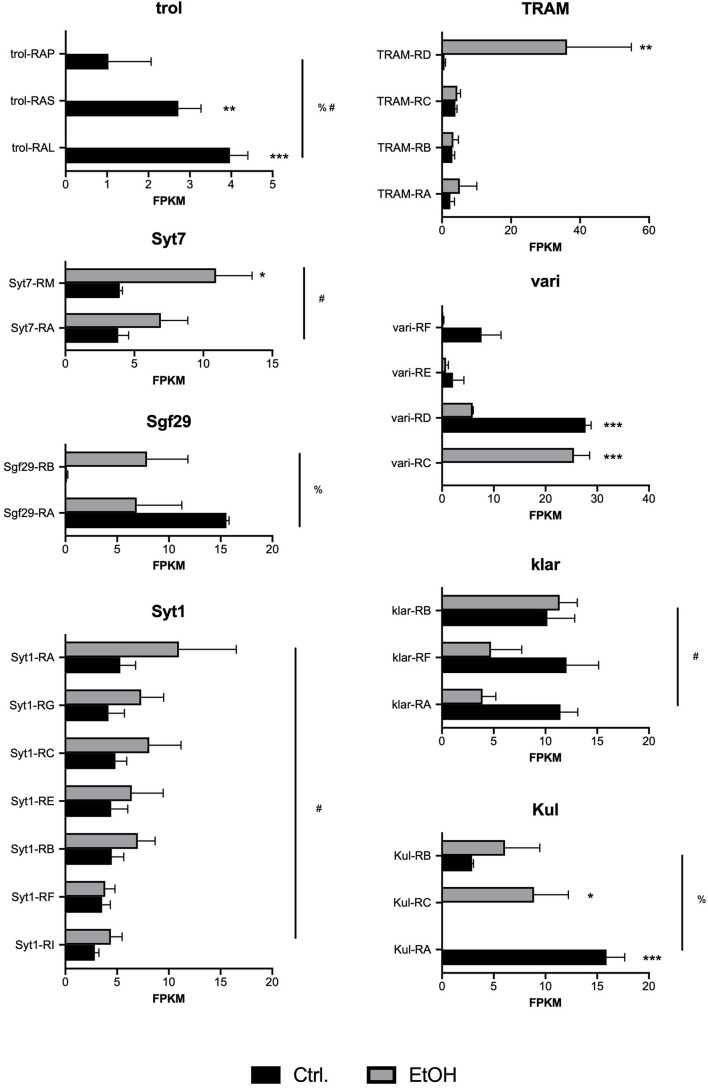
Transcript-specific expression induced by chronic alcohol exposure. Shown are FPKM counts in control (Ctrl, black bars) and alcohol treated (EtOH, gray bars) larvae for independent transcriptional isoforms of eight selected genes: *trol*, *Syt7*, *Sgf29*, *Syt1*, *TRAM*, *vari*, *klar*, and *Kul*. Significant differences were detected by Two-way ANOVA followed by Sidak’s multiple comparisons test. Asterisks denote significant differences between control and alcohol treated samples (*p < 0.05, **p < 0.01, ***p < 0.001). The pound sign (#) denotes a significant effect of treatment on all transcripts within the gene (p < 0.05). The percent (%) sign denotes a significant interaction between treatment and transcript identity (*p* < 0.05).

It is important to note that none of the differentially expressed transcripts reach a *q*-value (FDR) < 0.05. Unfortunately, this is a common problem associated with the larger number of multiple corrections used in transcript-level analyses as compared with gene-level analyses. Analysis of individual transcripts results in more than 34,000 multiple corrections, as compared to gene-level analysis, where only there are approximately 16,000 multiple corrections. However, we believe that examination at the transcript level is particularly powerful as it detects changes that affect single transcriptional isoforms instead of whole gene transcription. This is important since most *Drosophila* genes can produce up to 10 distinct transcripts and 5 different protein products (Brown et al., 2014), and thus important changes can be obscured by opposing transcriptional responses of different transcripts within the same gene. Therefore, we proceeded with a statistical assessment of differential transcript usage of a selected list of implicated targets (Figure 2). This analysis reveals several genes where expression of specific transcriptional isoforms within a gene are significantly changed, while others remain unchanged. This was the case of the genes *TRAM*, *klar*, *Syt1*, *Syt7*, and *trol.* In other cases, however, as described before, some transcriptional isoforms were significantly upregulated by alcohol, while other transcriptional isoforms within the same gene were significantly downregulated. As shown in Figure 2, this was the case of the genes *Sgf29*, *vari*, and *Kul*, where alcohol differentially affects distinct transcriptional isoforms.

Finally, to identify potential biological pathways involved in the neuroadaptation to alcohol, we performed GO enrichment analysis of the differentially expressed genes. This analysis was completed using the Database for Annotation, Visualization and Integrated Discovery (DAVID) tool (Huang da et al., 2009). GO term enrichment analysis among the upregulated transcripts revealed important biological processes involved (Table 2). The most significantly enriched GO term was “histone H3 acetylation,” with a fold enrichment of 20.8. The genes associated with this term include *CG6907*, *Sgf29*, and *trx*, which are well-known chromatin remodelers involved in the positive regulation of transcription and biological development.

**TABLE 2:** Overrepresented gene ontology (GO) annotations for alcohol-upregulated genes.

**Rank**	**Enriched gene ontology terms (upregulated genes)**	**Fold enrichment**	**Fisher exact score**	**Genes**
1	Histone H3 acetylation	20.80	3.90E-04	*CG6907*, *Sgf29*, *trx*
2	Regulation of calcium ion-dependent exocytosis	60.09	4.50E-04	*Sec23*, *Ank*
3	Protein targeting to plasma membrane	60.09	4.50E-04	*Syt1*, *Syt7*
4	Calcium ion-regulated exocytosis of neurotransmitter	45.07	8.30E-04	*Syt1*, *Syt7*
5	Response to hyperoxia	40.06	1.10E-03	*CG33129*, *Tm1*
6	Basement membrane organization	36.05	1.30E-03	*Sec23*, *Pten*
7	Larval chitin-based cuticle development	22.53	3.50E-03	*Sec23*, *Lcp9*
8	Endoplasmic reticulum organization	21.21	3.90E-03	*Sec23*, *Rtnl1*
9	Regulation of establishment of planar polarity	21.21	3.90E-03	*Akap200*, *Pten*
10	Positive regulation of dendrite morphogenesis	18.97	4.90E-03	*Sec23*, *G9a*
11	Chemical synaptic transmission	8.19	5.80E-03	*CaMKII*, *sky*, *Syt1*
12	Long-term memory	7.51	7.40E-03	*CaMKII*, *spoon*, *G9a*
13	Neurotransmitter secretion	6.08	1.30E-02	*Syt1*, *nSyb*, *Syt7*
14	Sensory perception of pain	2.40	1.70E-02	*CtBP*, *CaMKII*, *Akap200*, *stj*, *trx*, *rn*, *TRAM*, *CG5757*

*Gene ontology annotations within the “biological process” category that were enriched in alcohol-upregulated genes were identified using DAVID. For each term, fold enrichment, fisher exact scores, and the names of associated genes identified are listed.*

Interestingly, within the most significant GO terms enriched amid upregulated genes, we also found several terms associated with the regulation of synaptic transmission. Among these, we find “regulation of calcium ion-dependent exocytosis” (represented by *Sec23* and *Ank*), “calcium ion-regulated exocytosis of neurotransmitter” (represented by *Syt1* and *Syt7*), “chemical synaptic transmission” (represented by *CaMKII*, *sky*, and *Syt1*), and “neurotransmitter secretion” (represented by *Syt1*, *nSyb*, and *Syt7*). This suggests that alcohol may be causing dysregulation of chemical synaptic transmission in neurons across the brain, a phenomenon known to occur when individuals are continuously exposed to alcohol (Kyzar and Pandey, 2015). Other important categories also represented include “long-term memory” (represented by *CaMKII*, *spoon*, and *G9a*) and “sensory perception of pain” (represented by *CtBP*, *CaMKII*, *Akap200*, *stj*, *trx*, *rn*, *TRAM*, and *CG5757*). Not surprisingly, several genes are represented in different GO terms, suggesting that the terms belong to interconnected biological processes.

Within the down-regulated transcripts, GO term enrichment analysis also revealed important biological processes (Table 3). This time, the top enriched GO term was “specification of segmental identity, thorax,” with a fold enrichment of 91.63. The genes associated with this term include *Antp* and *Ubx*, two well-studied genes involved in biological development. In fact, many other GO terms were also associated with biological development and cell fate specification, suggesting a systematic downregulation of development. On the other hand, we again observed genes important for “sensory perception of pain,” however this time, the genes represented were different (*P5CDh1*, *dom*, *fok*, *Dad*, *UbcE2H*, *Gp150*, *CG4975*, *VhaPPA1-1*, *Unr*, *fax*, *Iswi*, *Vha100-1*) with one exception: *TRAM*—this gene had one upregulated transcriptional isoform, and one downregulated transcriptional isoform. The fact that this GO-term appears in both the upregulated and downregulated gene cohorts suggests that the genes involved in this process serve as an important regulatory switch.

**TABLE 3:** Overrepresented gene ontology (GO) annotations for alcohol-downregulated genes.

**Rank**	**Enriched gene ontology terms (downregulated genes)**	**Fold enrichment**	**Fisher exact score**	**Genes**
1	Specification of segmental identity, thorax	91.63	1.60E-04	*Antp*, *Ubx*
2	Sensory perception of pain	2.97	3.70E-04	*P5CDh1*, *dom*, *fok*, *Dad*, *UbcE2H*, *TRAM*, *Gp150*, *CG4975*, *VhaPPA1-1*, *Unr*, *fax*, *Iswi*, *Vha100-1*
3	Positive regulation of gene silencing by miRNA	45.82	7.70E-04	*Sdc*, *dom*
4	Open tracheal system development	6.74	8.60E-04	*Ubx*, *vari*, *grh*, *shot*, *spi*
5	Muscle cell fate specification	34.36	1.40E-03	*Antp*, *Ubx*
6	Negative regulation of transcription, DNA-templated	5.73	1.80E-03	*rhea*, *Bin1*, *Gug*, *Iswi*, *ph-p*
7	Terminal branching, open tracheal system	11.45	2.20E-03	*VhaPPA1-1*, *rhea*, *Vha100-1*
8	Muscle attachment	11.14	2.40E-03	*Tsp*, *rhea*, *shot*
9	Muscle organ development	6.62	3.10E-03	*Tsp*, *Sdc*, *Iswi*, *Dys*
10	Vacuolar acidification	22.91	3.30E-03	*VhaPPA1-1*, *Vha100-1*
11	Larval somatic muscle development	8.09	6.00E-03	*rhea*, *akirin*, *Gug*
12	Motor neuron axon guidance	6.25	1.20E-02	*Mp*, *Sdc*, *trol*
13	Transcription, DNA-templated	2.56	1.90E-02	*dan*, *dom*, *Bin1*, *Dad*, *Ubx*, *grh*, *Iswi*
14	Positive regulation of transcription from RNA polymerase II promoter	3.01	2.50E-02	*akirin*, *Antp*, *lok*, *grh*, *Iswi*

*Gene ontology annotations within the “biological process” category that were enriched in alcohol-downregulated genes were identified using DAVID. For each term, fold enrichment, fisher exact scores, and the names of associated genes identified are listed.*

## Discussion

RNA sequencing analysis of the effects of chronic alcohol exposure on the *Drosophila* larval brain showed many interesting differentially expressed genes. Our findings indicate that alcohol is altering different brain functions and disrupting several developmental pathways, many of them involved in higher-order processing. We find of particular interest the gene cohorts involved in the regulation of synaptic transmission, learning and memory, as well as those involved in the epigenetic control of developmental processes.

One of the most interesting gene cohorts seen in our data lies within the family of synaptic calcium-binding SNARE proteins, Synaptotagmins. This protein family is incredibly important in the process of vesicle trafficking and neurotransmitter release, both in *Drosophila* and mammals (Quiñones-Frías and Littleton, 2021). Our results show two Synaptotagmin paralogs, Syt1 and Syt7. Syt1 has been implicated in alcohol-related memory encoding in the *Drosophila* mushroom bodies (Petruccelli et al., 2020). Interestingly, Syt1 interacts closely with nSyb, a vesicle fusion protein also over-expressed in our data (Wang and Chapman, 2010), strengthening the notion that chronic alcohol affects synaptic integrity. In the case of Syt7, an interaction exists between said isoform and the synaptic protein Drep2 (Andlauer et al., 2014). This protein was present in our results, though not significantly regulated. Drep2 is known to be involved in learning and memory in *Drosophila* and is also known to colocalize with metabotropic glutamate receptors (Andlauer et al., 2014). Another study conducted in mice revealed *Syt7*’*s* presence in glutamatergic hippocampal neurons (Weber et al., 2014). During larval development, Syt7 is found in the neuronal cell bodies, which could explain the interaction with Drep2, a postsynaptic protein (Adolfsen et al., 2004; Andlauer et al., 2014). How these proteins interact with each other during the adult stages of the *Drosophila*, when Syt7 is localized in the synapse, requires further investigation (Guan et al., 2020). This interaction of the significantly over-expressed gene *Syt7* with *Drep2* is suggestive of an altered signaling pathway or mechanism as a result of chronic alcohol exposure.

Similarly, we found that many transcripts of genes involved in histone modifications and chromatin remodeling were altered as a result of chronic alcohol exposure. Epigenetic modifications are essential regulators of the transcriptional adaptation to drugs of abuse. In the past years, studies have addressed many epigenetic mechanisms important for modulating neural physiology as a response to alcohol (Ghezzi et al., 2013, 2017; Ramirez-Roman et al., 2018). Our results strengthen this idea and reveal many potential regulatory elements relevant to this process. As previously mentioned, we observed significant upregulation of genes involved in histone acetylation and methylation, including *Sgf29*, *G9a*, and *trx*. The protein Sgf29 is part of the Ada2/Gcn5/Ada3 transcription activator complex. However, little is known about its biological function (Petruk et al., 2006; Suganuma et al., 2008). On the other hand, G9a does not only function as a histone modification protein but is also involved in learning and memory, suggesting a tight link between the epigenetic reprogramming induced by alcohol and the modulation of higher-order processes in the brain (Kramer et al., 2011). Finally, Trx, a chromatin-modifying enzyme involved in gene regulation, is known to antagonize the epigenetic silencing by Polycomb group proteins and is known to contribute to axon guidance, eye development, and germ cell migration. Given that all these proteins are involved in histone modifications or chromatin remodeling, our results strengthen the idea that these mechanisms are of utter importance to cognitive alcohol neuroadaptations.

Another interesting phenomenon induced by chronic alcohol exposure identified in this study is the presence of potential transcriptional switches. For a subset of genes, differential transcript usage was triggered by alcohol, where one specific isoform was upregulated while another was downregulated. One particularly significant example of this phenomenon is observed in the gene *vari*, where the transcriptional isoform *vari-RC* is significantly induced by alcohol, while the isoform *vari-RD* is suppressed (Figure 2). Close inspection of the *vari* locus reveals that these two major transcript isoforms share seven exons encoding the core PDZ, SH3, HOOK, and GUK domains. However, the longer isoform (*vari-RD*) also encodes an N-terminal L27 protein-protein interaction domain that is absent from the shorter isoforms (*vari-RC*). The protein encoded by *vari* is a membrane-associated guanylate kinase (MAGUK) involved in tracheal system development and septate junction assembly (Wu et al., 2007). Vari proteins are known to interact with several proteins, including Fasciclin 3 (Wells et al., 2013), Semaphorin 1a (Jeong et al., 2017), Neurexin IV, and veli (Bachmann et al., 2008); these proteins are involved in ensheathing glial cells and neurons, axon guidance and synaptic growth at the neuromuscular junction, respectively. The differential expression of two Vari isoforms, which differ by the presence or absence of the L27 domain, is certainly intriguing. L27 domains can potentially stabilize interactions to different proteins by binding to the L27 domain of a corresponding partner. However, its role in alcohol neuroadaptation remains to be resolved.

While our study centers primarily on the transcriptional changes, additional experiments focusing on morphological and electrophysiological changes should offer new insights into the neuroadaptive mechanisms of chronic alcohol exposure. Nonetheless, this study facilitates our understanding of molecular and cellular dynamics of alcohol neuroadaptation in *Drosophila* larvae. Findings from this study, along with future research, could also lead to a better understanding of the effect of alcohol on learning and memory beyond the fruit fly and gain an insight into how addiction to this substance may contribute to cognitive decline and dependence in humans. Alcohol misuse is, in fact, one of the leading causes of death in the United States, with approximately 95,000 deaths occurring annually. According to the National Institute on Alcohol Abuse and Alcoholism, in 2018, 14.1 million adults of ages 18 and up suffered from alcoholism (NIAAA, 2020).

## Data Availability Statement

The data presented in this study are deposited in the NCBI Gene Expression Omnibus (GEO) data repository, accession number GSE185625.

## Author Contributions

AA-G and AG designed the study and wrote and reviewed the manuscript. AA-G and JA-G performed the alcohol treatments, larval brain extractions, and prepared samples for sequencing. CI-H and NF-U assisted in the larval brain dissections. AA-G, AM-M, and AG performed the bioinformatic analysis. All authors contributed to the article and approved the submitted version.

## Conflict of Interest

The authors declare that the research was conducted in the absence of any commercial or financial relationships that could be construed as a potential conflict of interest.

## Publisher’s Note

All claims expressed in this article are solely those of the authors and do not necessarily represent those of their affiliated organizations, or those of the publisher, the editors and the reviewers. Any product that may be evaluated in this article, or claim that may be made by its manufacturer, is not guaranteed or endorsed by the publisher.

## Supplementary Material

Supplementary Table 1 Complete annotated list of differentially expressed transcripts after chronic alcohol exposure.

Supplementary Table 2 Complete FPKM counts for each replicate sample.
